# Anticancer and Immunogenic Properties of Cardiac Glycosides

**DOI:** 10.3390/molecules22111932

**Published:** 2017-11-08

**Authors:** Naira Fernanda Zanchett Schneider, Claudia Cerella, Cláudia Maria Oliveira Simões, Marc Diederich

**Affiliations:** 1 Programa de Pós-Graduação em Farmácia, Centro de Ciências da Saúde, Universidade Federal de Santa Catarina, Florianópolis SC 88040-900, Brazil; nairafzs@gmail.com (N.F.Z.S.); claudia.simoes@ufsc.br (C.M.O.S.); 2 Laboratoire de Biologie Moléculaire et Cellulaire du Cancer (LBMCC), Hôpital Kirchberg, 9, rue Edward Steichen, 2540 Luxembourg, Luxembourg; claudia.cerella@lbmcc.lu; 3 Department of Pharmacy, Research Institute of Pharmaceutical Sciences, College of Pharmacy, Seoul National University, Building 29 Room 223, 1 Gwanak-ro, Gwanak-gu 08826, Korea

**Keywords:** cardiac glycosides, anticancer, immunogenic cell death, cytotoxic effects, cancer cells, Na/K-ATPase

## Abstract

Cardiac glycosides (CGs) are natural compounds widely used in the treatment of several cardiac conditions and more recently have been recognized as potential antitumor compounds. They are known to be ligands for Na/K-ATPase, which is a promising drug target in cancer. More recently, in addition to their antitumor effects, it has been suggested that CGs activate tumor-specific immune responses. This review summarizes the anticancer aspects of CGs as new strategies for immunotherapy and drug repositioning (new horizons for old players), and the possible new targets for CGs in cancer cells.

## 1. Introduction

Drugs directly or indirectly derived from natural products are used in oncology and play an important role in this field. For example, the vinca alkaloids, vinblastine and vincristine, are isolated from *Catharanthus roseus* L., Apocynaceae, and are used to treat leukemia, lymphoma, testicular cancer, breast cancer, lung cancer, and Kaposi’s sarcoma. Another example is paclitaxel, which is obtained from *Taxus brevifolia* Nutt., Taxaceae, and is significantly active against ovarian, breast, and lung cancer [1].

Among natural products, cardiac glycosides (CGs), mainly those obtained from *Digitalis* spp., are traditionally used in the treatment of congestive heart failure and some arrhythmias. They act by inhibiting the Na/K-ATPase pump. Recently these compounds exhibited in vitro cytotoxic and cytostatic effects against various human cancer cell lines, which is attributable to their ability to induce cell-type-specific cell death modalities [2]. Additionally, selected CGs entered phase I and II clinical trials for the treatment of solid tumors with satisfactory safety and efficacy [3,4].

CGs have been used for the treatment of heart disease for over 200 years; in 1785, the physician William Withering published a book entitled “*An account of the foxglove and its medical uses*” [5]. CGs were later characterized by their high specificity and powerful action on the cardiac muscle. Therefore, they were used for the treatment of congestive heart failure and administered in combination with diuretics to treat atrial fibrillation. They were also recommended for the prophylaxis and treatment of some arrhythmias, such as paroxysmal atrial tachycardia and cardiogenic shock.

## 2. Cardiac Glycosides

**Figure 1:**
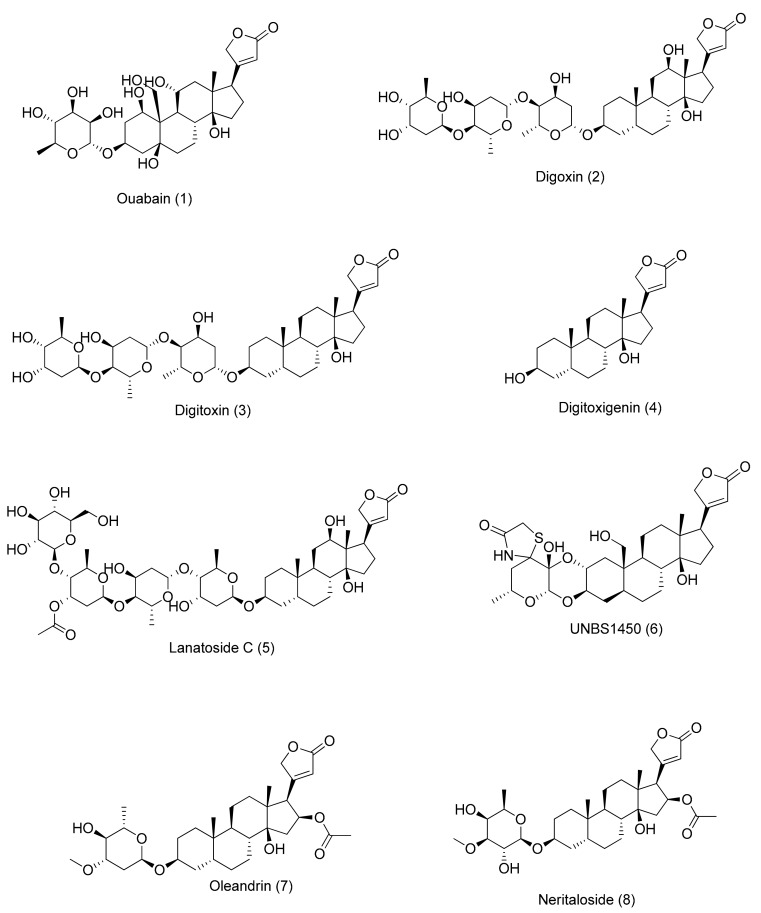
Molecular structures of cardenolides, as described in the text. Each molecule is numbered in order of its appearance in the text.

Cardiac glycosides are found in some plant families, such as Apocynaceae (*Asclepias* sp. L. and *Nerium oleander* L.) and Plantaginaceae (*Digitalis lanata* Ehrh. and *Digitalis purpurea* L.) [6,7] as well as in animals (bovine hypothalamus) [8]. Some studies have reported that humans can produce endogenous substances similar to ouabain (**1**) [9] and digoxin (**2**) [10] (Figure 1). For example, endogenous digoxin-like immunoreactive factor (DLIF) was found in human blood plasma. The selective induction of apoptosis by DLIF and ouabain-like factor, both secreted by the adrenal cortical glands, is believed to regulate the activity of Na/K-ATPase. Regarding the induction of apoptosis, DLIF is at least 100-fold more potent than digoxin or ouabain in Jurkat cells. Furthermore, other research groups have shown that cholesterol and progesterone are substrates for the biosynthesis of endogenous CGs, which is controlled by hormones, such as renin–angiotensin, endothelin, and adrenaline [11,12].

### 2.1. Chemical Structure

Regarding their chemical structure, CGs contain a steroid nucleus with an unsaturated lactone moiety at C17 and a sugar moiety bound to C3. The nature of this lactone moiety characterizes two subgroups: cardenolides (five-membered α,β-unsaturated butyrolactone ring) and bufadienolides (six-membered α,β-γ,δ-unsaturated 2-pyrone ring). A/B and C/D rings are in *cis* conformation, whereas B/C rings are fused in *trans* conformation [13]. The subgroup of cardenolides is characterized by the aglycones, such as digitoxigenin, digoxigenin, gitoxigenin, gitaloxigenin, diginatigenin, oleandrigenin, uzarigenin, xysmalogenin, and canarigenin [14]. A wide variety of sugars may be linked to the aglycone moiety. The most common sugars are digitoxose, oleandrose, diginose, fructose, rhamnose, and glucose. Although sugars are not directly responsible for the cardiotonic activity of CGs, they affect pharmacodynamics and pharmacokinetics of this class of compounds. For example, aglycones are absorbed faster and metabolized more easily than glycosylated CGs [15]. However, the presence of sugars improves their solubility and distribution in the body. In addition, the stereochemistry of the sugar influences its binding affinity to the receptor protein. The most studied CGs are ouabain (**1**), digoxin (**2**), digitoxin (**3**), digitoxigenin (**4**), and lanatoside C (**5**). From a therapeutic point of view, the most important compounds are digoxin (**2**) and digitoxin (**3**) [16].

### 2.2. Cardiotonic Activity

The cardiotonic action occurs through inhibition of Na/K-ATPase, which promotes cardiac muscle contraction [17]. When Na/K-ATPase is inhibited, the influx of Na^+^ increases and that of K^+^ decreases in myocytes. This ion exchange requires ATP. The intracellular Na^+^ modulates the activity of a membrane carrier involved in the exchange of Ca^2+^ by Na^+^, promoting an increase in the intracellular levels of Ca^2+^ by influx or mobilization of the sarcoplasmic reservoirs. An increase in Ca^2+^ ions increases cardiac contractile force since more Ca^2+^ is available for the contractile proteins. In the relaxed heart, tropomyosin masks the binding site of myosin through a steric blockage. When the heart muscle contracts, Ca^2+^ interacts with troponin resulting in a conformational change of tropomyosin, which unmasks the myosin binding site and enables the formation of the actin–myosin complex, thereby inducing ATP-dependent myocardial contraction. Therefore, the increase in intracellular Ca^2+^ levels, triggered by CGs, causes an increase in muscle tone and circulating blood volume per minute and reduces heart rate and stroke volume [18].

Despite the extensive use of CGs as positive inotropic agents, investigation into the effects of these compounds in other pathological conditions has intensified in recent years, presenting new therapeutic possibilities, including anticancer activity [15].

## 3. Anticancer Activity

### 3.1. Early Observations

The first evidence of the in vitro cytotoxic effects of CGs against human tumor cells and their antitumor action in vivo was reported in the late 1960s [19]. Twenty years later, published reports about these compounds still attract researcher’s attention [20,21,22]. For more detailed historical aspects of investigations about CGs, refer to the following reviews: [15,23]. These studies triggered further mechanistic investigations about the cytotoxic, antitumor, and anticancer potential of CGs.

### 3.2. Repurposing of Approved Drugs

Repurposing of approved drugs to treat diseases other than those for which they were developed has been used in different clinical areas. This process offers a cost-effective and fast way to develop new treatments using drugs that have already been proven safe in humans [24]. Recently, Varbanov et al. (2017) performed an in vitro screening using A549 (lung cancer) and PANC-1 (pancreatic carcinoma) cells and tested 1280 chemically and pharmacologically diverse compounds from the Prestwick Chemical Library^®^ using a fluorescent-based cell viability assay. More than 100 compounds were identified as hits in one or both cell lines, and the authors found that the promising candidates for repositioning that emerged from this study included CGs (digoxin, lanatoside C, proscillaridin A, digoxigenin, and digitoxigenin) [25]. A two-stage transdisciplinary approach, including a high-throughput laboratory-based screening and a large prospective cohort study, was used to identify possible anti-prostate cancer drugs [26]. In stage one, 3187 FDA-approved drugs were screened in androgen-dependent and -independent prostate cancer cells, and digoxin was identified as the most potent inhibitor of cell proliferation. Thereafter, in stage two, the association between digoxin and prostate cancer risk in 47,884 men followed for twenty years (1986–2006) was evaluated. The authors found that regular digoxin users, especially those who had used digoxin for ≥10 years, had a lower prostate cancer risk. They concluded that digoxin was highly potent for inhibiting prostate cancer cell growth in vitro and its use was associated with a 25% lower prostate cancer risk [27]. Nevertheless, it is important to mention that some clinical evidences suggest that the doses of CGs required for cancer treatment may be lower than those used in cardiac patients [27].

### 3.3. Primary Targets of CGs

After these initial reports, several studies were published regarding the antiproliferative activity of plant extracts containing CGs, semisynthetic CGs, and natural CGs isolated from plants or obtained by fungus biotransformation. The effects of these CG preparations on different types of cancer cell lines, including those of breast cancer, prostate cancer, pancreatic cancer, leukemia, neuroblastoma, and melanoma were described [13,28].

Despite the numerous studies performed with these compounds and extracts, the detailed mechanism by which they act on tumor cells is still being investigated. Many theories have been proposed and several intracellular intermediates and independent molecular pathways have been described as modulators of the observed effects. However, none of these theories sufficiently describes the mechanism of action since it is extremely complex. In addition, the heterogeneity of the proposed mechanisms was thought to be a consequence of the specific cell types against which the compounds were tested [2,29].

One of the most promising theories related to the primary target of CGs in tumor cells is that CGs bind to the alpha subunit of Na/K-ATPase. This interaction leads to changes in pumping activity, which increases intracellular Na^+^ levels and depletes K^+^ levels. Consequently, the intracytoplasmic Ca^2+^ levels increase owing to exacerbation of mitochondrial Na^+^/Ca^2+^ exchange. Moreover, CGs may also affect the non-pumping functions of Na/K-ATPase leading to the activation of intracellular signaling cascades. Beyond the Na/K-ATPase, CGs may interact with the plasma membrane via the steroid nucleus, thereby modifying membrane fluidity and indirectly affecting the function of several membrane proteins and receptors. Moreover, direct interaction and binding to other transmembrane receptors is plausible along with the ability to internalize and interact directly with potentially susceptible intracellular targets [2,30].

The CG binding site has been extensively discussed to understand how the sodium pump, which is composed by multifunctional groups of α and β subunits, is inhibited. The most important part of the binding site is represented by the first extracellular α-subunit channel [31,32]. Four α (α1–4) and three β (β1–3) isoforms have been characterized in mammals and their distribution depends on the cell type. All possible α and β combinations result in catalytically competent enzymes indicating that multiple Na/K-ATPase isoenzymes can operate in cells [33].

It is important to emphasize that the α-1 subunit is overexpressed in some types of cancer, including non-small cell lung cancer (NSCLC), renal carcinoma, glioma, and melanoma, whereas the α-3 subunit is overexpressed in colon carcinoma [33,34,35]. In addition, in mice, α-2, α-3, and α-4 isoforms are naturally susceptible to CGs. In rodents, the α-1 isoform is resistant to the binding of ouabain. In humans, the situation is different because the α-1 isoform is sensitive to CGs and may therefore play a key role in the signal transduction pathways [36].

In this sense, targeting the Na^+^/K^+^ pump may be useful for treating patients with lung cancer. Inhibition of ATPase Na^+^/K^+^ Transporting Subunit Alpha 1 (ATP1A1) was recently investigated by treating STK11 mutant lung cancer cells with CGs. The STK11 (LKB1) mutation is a major mediator of lung cancer progression and a targeted therapy has not been implemented because STK11 mutations cause loss of function. Some CGs, such as digoxin, digitoxin and ouabain, directly inhibited ATP1A1 and exhibited selective antitumor effects in STK11 mutant lung cancer cells. Therefore, the STK11 (LKB1) mutation seems to be a novel biomarker for CGs, and may be a promising future research target [37].

Yang et al. investigated the intracellular distribution of the Na/K-ATPase α-3 subunit and showed that the intracellular location of this isoform is altered in human cancer cells, when compared to normal cells [38]. The effects of oleandrin (**7**) on extracellular regulated kinase (ERK) phosphorylation, cell proliferation and death, were investigated in differentiated and undifferentiated human colon cancer Caco-2 cells and in surgically resected specimens of normal and malignant colorectal and lung tissues. The Na/K-ATPase α-3 isoform was primarily located near the cytoplasmic membrane in normal human colon and lung epithelia; however, the expression of this subunit in their paired cancer epithelia was shifted to a peri-nuclear position. Similarly, the α-3 isoform was translocated from a cytoplasmic membrane location in differentiated Caco-2 cells to a peri-nuclear location in undifferentiated Caco-2 cells. Interestingly, oleandrin (**7**) exerted stronger antiproliferative activity in undifferentiated Caco-2 cells than in differentiated cells. As a result, this compound caused autophagic cell death and altered ERK phosphorylation in undifferentiated, but not differentiated, Caco-2 cells. Altogether, changes in the α-3 cellular position may become potential targets for cancer therapy [39].

### 3.4. Signal Transduction Pathways Triggered by CGs

In addition to identifying the primary target, multiple signaling pathways that are potentially activated or inhibited by CGs have been evaluated. From previous studies, some targets have been proposed, including ionic disturbances and changes in intracellular levels of Na^+^ and K^+^ ions, which can generate harmful effects and alter the conformation of many proteins [2,40]; inhibition of DNA topoisomerase II [41]; inhibition of the nuclear factor-kappa-light-chain-enhancer of activated B cells (NF-κB)-mediated pathways, which are constitutively active in many types of cancer [28,42]; changes in cell cycle, specifically blocking S and G2/M phases [43,44]; inhibition of interleukin (Il)-8 production [45]; and modulation of the myeloid cell leukemia-1 (Mcl-1) as an essential factor for cell death [2,28,46].

Furthermore, the effects of CGs in several stages of the metastatic cascade were reported. Ouabain (**1**) inhibited migration of H292 lung tumor cells via the suppression of regulatory migration proteins, such as focal adhesion kinase (FAK) and Akt [16]. Therapeutic concentrations of digitoxin (**3**) inhibited angiogenesis in human umbilical vein endothelial cells (HUVEC) and promoted FAK activation by several pro-angiogenic stimuli [11]. Convallatoxin (**11**) inhibited migration and invasion via regulation of metalloproteinases (MMP)-2 and -9 [47], and inhibited angiogenesis in vitro and in vivo [48]. Gamabufotalin (**12**) inhibited vascular endothelial growth factor (VEGF)-triggered HUVEC proliferation, migration, invasion, and tubulogenesis in vitro by suppressing the VEGF receptor-2 signaling pathway [49]. Additionally, the anti-migratory effects of several CGs in breast cancer cells were related to the inhibition of the Na^+^/K^+^ ion exchange [50]. Additionally, beyond these well-established pathways and intermediate targets, other mechanisms related to the mode of action of CGs are shown in Table 1.

The effects of CGs on the ion exchange by the Na^+^/K^+^-ATPase have been investigated extensively. However, over the last 10 years, several studies determined that cells provide normal survival signals [30,40,51] and signs of death in tumor cells [52] when CGs bind to Na^+^/K^+^-ATPase. To explain this fact, the presence of two types of Na^+^/K^+^ channels in the membrane was suggested [51]. In addition to the classic channel for energetic ion transduction, a second category of Na^+^/K^+^ channel restricted to the caveolae (small lipid invaginations in the cells) forms, the so-called Na^+^/K^+^-ATPase signalosome, was described. Furthermore, it is believed that the signaling domain of this type of Na^+^/K^+^-ATPase is located at membrane depressions of clathrin on the cytoplasmic side. During the binding of CGs, conformational changes in the structure of the protein trigger a cascade of signal transduction pathways [36]. These pathways are initiated by the interaction of CGs to Na^+^/K^+^-ATPase to activate Src tyrosine kinase, which in turn activates the epidermal growth factor receptor (EGFR). The activated EGFR recruits and stimulates SHC, growth factor receptor-bound protein 2 (GRB2), and SOS adapter proteins, leading to the activation of Ras and MAPK cascades. In parallel, phospholipase C (PLC) and inositol triphosphate (IP3) allow Na^+^/K^+^-ATPase to interact with the endoplasmic reticulum. This stimulation results in a temporary or repeated release of Ca^2+^ into the cytoplasm. The perturbation of Ca^2+^ homeostasis was described to induce signaling pathways, leading to activation of NF-κB [2,28].

Additionally, targeting of epigenetic pathways may also be a promising approach for cancer therapy and some CGs seem to act in this way. Targeting Ca^2+^ signaling may indeed reverse epigenetic silencing of tumor suppressor genes (TSGs). A recent screening discovered CGs as particularly able to reactivate silenced genes in colon cancer cells. Ouabain (**1**), lanatoside C (**5**), digoxin (**2**), digitoxin (**3**), and proscillaridin (**14**) were shown to alter Ca^2+^ signaling and trigger Ca^2+^-calmodulin kinase (CamK) activity, leading to nuclear exclusion of methyl CpG binding protein (MeCP)2. The alteration of Ca^2+^ signaling results in activated CamK, which plays a central role in TSGs reactivation and cancer cell death. These findings indicated that targeting Ca^2+^ fluxes is essential for the regulation of the epigenetic mechanism of action. Altogether, Ca^2+^ signaling was identified as a novel pathway that can be targeted to reactivate TSGs in cancer [53].

As showed so far, many of the anti-cancer activities of CGs have been linked to the inhibition of the Na^+^/K^+^-ATPase also called “on-target” effects. However, some studies indicated additional intracellular targets for CGs that could be unrelated to Na^+^/K^+^-ATPase-mediated ion exchange, called “off-target” effects. Here the endosomal trafficking of both Na^+^/K^+^-ATPase and Src kinase, and the downregulation of pro-survival Mcl-1 and Bcl-xL pro-survival proteins could belong to this regulatory category. Firstly, the endosomal trafficking of Na^+^/K^+^-ATPase subunits can be internalized into endosomes by a lipid raft- and clathrin-coated vesicle mechanism, culminating in the formation of recycling and late endosomes culminating in endolysosomes and leading to cell death [13]. Na^+^/K^+^-ATPase α1 internalization was triggered by ouabain (**1**) in the human lung carcinoma cell line H1299. The α1 internalization is not the result of changes of the cellular ion balance but probably is triggered by a conformational change of the protein itself [54]. Src is a non-receptor tyrosine kinase that is associated with the Na^+^/K^+^–ATPase transmembrane complex in its active form, it co-localizes with endosomal markers, and associates with the tubule-organizing center (TOC) in its inactive form. The activation of endosomal trafficking by CGs could represent an essential activator of Src intracellular transport, as membrane targeting of Src intimately depends on endosomal delivery to the plasma membrane. Secondly, it was described that some cardenolides [46,55] and bufadienolides [46,56] downregulate, in several cancer cell lines, one anti-apoptotic protein, Mcl-1, a Bcl-2 family member responsible for cancer cell resistance, and consequently could be considered as an essential and universal target of CGs. The cardenolide UNBS1450 abrogates anti-apoptotic Mcl-1 expression leading to the recruitment of pro-apoptotic Bak and Bax proteins in leukemia cells. The authors showed that Mcl-1, but not Bcl-xL or Bcl-2, was downregulated prior to induction of apoptosis. Even if CGs modulate the expression of several Bcl-2 family proteins, their strongest and most ubiquitous effect in cancer cell lines was the modulation of Mcl-1 [46]. The bufadienolide bufalin induced apoptosis in NSCLC H1975 cells in a caspase-dependent manner, downregulating Mcl-1 expression. In these cells, bufalin did not reduce Mcl-1 mRNA expression, but strongly promoted Mcl-1 protein degradation [56].

### 3.5. Induction of Multiple Cell Death Modalities

As discussed so far, the interaction with the Na/K-ATPase and the activation of these intracellular signaling cascades may culminate in different types of cell death. It is already known that CGs can induce apoptosis, autophagy, anoikis and immunogenic cell death, depending on the cell type and chemical characteristics [40,46]. These multiple potential cytotoxic pathways are attracting the interest of researchers because they could provide alternative routes of treatment for tumors resistant to apoptosis-inducing agents [28]. The cardenolide UNBS1450 (**7**), already mentioned above, is an example of a CG that can induce apoptotic cell death in human leukemic cells [43], and autophagic cell death in human glioma, prostate, and NSCLC cell lines [33]. Recently, UNBS1450 was shown to induce a neuroblastoma cell-type-specific response leading to apoptosis or necroptosis. In neuroblastoma SH-SY5Y cells, this CG induced mitophagy and a ROS response, triggering an accumulation of autophagosomes, thereby mediating mitochondrial accumulation and causing apoptosis via oxidative stress-induced lysosomal destabilization. Interestingly, the induction of mitochondria autophagosomal clearance made stromal SK-N-AS neuroblastoma cells more resistant to UNBS1450 treatment, inducing necroptosis at high doses [68].

CGs, including oleandrin (**7**) [69,70], digitoxin (**3**) [71], digoxin (**2**) [72], ouabain (**1**) [73], lanatoside C (**5**) [74], and the cardenolides extracted from *Cerbera manghas* L. [25,75] and *Periploca graeca* L. [76], induced apoptotic cell death in different tumor cell lines. Some of these compounds induced the intrinsic apoptotic pathway characterized by BAK/BAX activation leading to release of cytochrome *c* and loss of membrane potential [43,77]. Other studies demonstrated the ability of these compounds to affect the extrinsic apoptotic pathway through upregulation of death receptors [78] or their ligands, such as the Fas ligand (FasL) [69].

Regarding autophagy, digoxin (**2**) and ouabain (**1**) induced autophagy in NSCLC [44] and oleandrin (**7**) induced autophagic cell death in human glioblastoma and pancreatic tumor cell lines [7]. These compounds also triggered an increase of autophagosome formation and lysosomal activity, both characteristic markers of this type of death. Moreover, the involvement of different key regulators in this process, such as ATG’s, LC3-BII, beclin-1 and mTOR was demonstrated [7].

### 3.6. Immunogenic Activity

**Figure 2:**
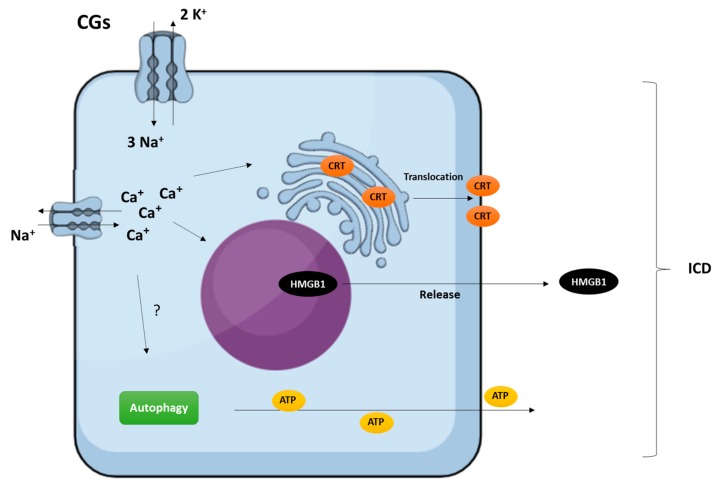
Overview of CG-induced immunogenic cell death (ICD) in vitro. Cancer cells treated with CGs release the hallmarks of ICD, exhibiting the endoplasmic reticulum chaperon calreticulin (CRT) on the outer leaflet of the plasma membrane, secreting ATP via an autophagy-dependent mechanism, and releasing high mobility group box 1 (HMGB1) danger-associated molecular pattern (DAMP) in a mechanism dependent on Na^+^/K^+^-ATPase inhibition and cCa^2+^ increase [3].

Another active field of investigation related to the potential of CGs as anticancer agents concerns their immunogenic activity. Compounds that induce cytotoxic effects, while stimulating an immune response against dead cell-associated antigens, are known as immunogenic cell death (ICD) inducers [13]. Acceptable ICD inducers may restore immunosurveillance by increasing the immunogenicity of malignant cells. In addition to this “on-target” effect, compounds can also directly affect immunostimulatory or immunosuppressive cellular pathways through off-target effects [79]. In this sense, Menger et al. [80] identified CGs as efficient ICD inducers. Their immunogenic effects were associated with the inhibition of Na/K-ATPase and the consequent alterations in intracellular Ca^2+^ homeostasis. Figure 2 shows an overview of CG-induced ICD in vitro [3].

More specifically, in a screening conducted using a fluorescent biosensor–based platform to identify molecules that potentially induce ICD, CGs were recognized for converting non-immunogenic cell death modalities into bona fide ICD [80]. In addition, CGs improved the antineoplastic effects of non-immunogenic chemotherapeutic drugs in immunocompetent mice, but not in immunodeficient ones. Additionally, it was found that, in a cohort of carcinoma patients who received CGs along with non-immunogenic chemotherapeutic drugs, the disease was improved compared to the control group, who received only chemotherapeutic drugs. In other words, CGs exert antineoplastic effects primarily by stimulating the immunogenicity of cell death [3,80]. Subsequently, the same automated screening platform previously used to identify ICD inducers was used with the 879 anticancer compounds of the National Cancer Institute (NCI) Mechanistic Diversity Set. The authors identified scillaren A (**13**), proscillaridin (**14**), lanatoside C (**5**), and digitoxigenin (**4**) as additional potential ICD inducers [81].

Following the same line of reasoning, CD4^+^CD25^+^Foxp3^+^ regulator T (T_reg_) cells received considerable attention owing to the immunosuppressive properties they have shown in vitro and in vivo. T_reg_ cells play an important role in tumor tolerance by suppressing antitumor immunity. A recent study conducted with the bufadienolide gamabufotalin (**12**) showed that, at low concentrations [non-toxic for PBMCs (8 ng/mL)], it efficiently reduced the percentage of T_reg_ cells in mitogen-activated PBMCs. In this way, gamabufotalin may be a promising candidate for use as an adjuvant therapeutic agent for manipulating T_reg_ cells to increase the efficacy of anticancer drugs and promote the immunological clearance of cancer cells [82].

## 4. Clinical Trials

Some CGs have been investigated for cancer treatment in phase I and II clinical trials. Among them Anvirzel™, a lyophilized aqueous extract of *Nerium oleander*; PBI-05204, a *Nerium oleander* extract prepared by supercritical CO_2_ extraction; the compound UNBS1450 (**6**), a semisynthetic cardenolide derived from 2″-oxovoruscharin (**10**), which was extracted from the tropical shrub *Calotropis procera* (Aiton) WT, Asclepiadaceae [83]; the well-known CG digoxin and HuaChanSu, a traditional Chinese medicine extracted from the *Bufo* toad [3].

The extract Anvirzel™ contains several CGs, such as oleandrin (**7**), neritaloside (**8**), and the aglycone, oleandrigenin (**9**). Two of these, oleandrin (**7**) and oleandrigenin (**9**), inhibited the catalytic activity of the Na/K-ATPase and inhibited the expression of fibroblast growth factor 2 (FGF-2) in prostate cancer cells [84,85]. A study was conducted to determine the maximum tolerated dose (MTD) and safety of this extract in patients with advanced solid tumors. The results indicated that Anvirzel™ could be administered safely at doses up to 1.2 mL/m^2^/day. A limiting toxic dose was not found, but its MTD was determined to be 0.8 mL/m^2^/day. Eighteen patients were enrolled and completed at least one cycle of three weeks of treatment [4]. Anvirzel™ was tested in phase I to treat solid tumors and NSCLC alone and in combination with carboplatin and docetaxel for NSCLC [3].

Oleandrin (**7**), one of the cardenolides present in Anvirzel^TM^ and PBI-05204 extracts, inhibited the α-3 subunit of the Na/K-ATPase, which is correlated with cell proliferation, and FGF-2. It also inhibited the NF-κB pathway and phosphorylation of Akt and p70S6K, attenuating mTOR activity [86]. A phase I study was completed with PBI-05204 and its MTD recommended for phase II trials was determined in addition to pharmacokinetic (PK) and pharmacodynamic (PD) parameters in patients with advanced cancer (cancers of the bladder, colon, rectum, fallopian tube, breast, and pancreas). Forty-six patients received eight different doses of the extract PBI-05204 (0.6 to 10.2 mg/day) and seven patients were stable for more than four months. In this study, the authors concluded that the extract PBI-05204 was well tolerated at the maximum dose tested (10.2 mg/day) with few common adverse effects and no cardiotoxicity [28,86]. In conclusion, this clinical trial determined the safety and the PK and PD parameters of PBI-05204 extract in patients with advanced cancer. The recommended dose for phase II was 0.2255 mg/kg [87]. Currently, PBI-05204 is undergoing a phase II trial for metastatic pancreatic adenocarcinoma (https://clinicaltrials.gov/ct2/show/NCT02329717).

As cited above, 2″-oxovoruscharin (**10**), a cardenolide with a dihydrothiazole ring (rare in CG molecules), was isolated from *Calotropis procera.* It exhibited potent in vitro cytotoxic effects and inhibited Na/K-ATPase activity [83]. The 2″-oxovoruscharin derivative, obtained by reduction of the formyl group to a hydroxymethyl group, was named UNBS1450 (**6**), and was more cytotoxic in vitro when compared to its parent compound. UNBS1450 showed in vitro antiproliferative activity with IC_50_ values ranging from 10 to 50 nM in 58 human tumor cell lines from 11 different histopathological types [83]. This compound induced the disruption of actin filaments and affected several signaling pathways after binding to Na/K-ATPase [33]. Based on these findings, UNBS1450 was used in a phase I clinical trial in the European Community for the treatment of patients with lymphomas and solid tumors, but the study was closed in 2011 [28].

Currently, 16 clinical trials with digoxin (**2**) are in progress: eight trials were completed, four are recruiting patients, and four are active (https://clinicaltrials.gov/ct2/results?cond=cancer&term=digoxin). Several cancer types have been investigated, including head and neck, prostate, breast and lung cancer; melanoma; advanced solid malignant tumors; Kaposi’s sarcoma; and acute myeloid leukemia (AML). Digoxin (**2**) has been tested as a single drug and in combination with chemotherapeutic and immunotherapeutic agents with different mechanisms of action. For example, patients with advanced head and neck squamous cell carcinomas have a poor prognosis and the clinical trial named NCT02906800 proposes to treat these conditions with the combination of digoxin and cisplatin because digoxin may potentiate the efficacy of cisplatin via induction of antitumor immunity. This study is currently recruiting participants and will be completed in 2019 (https://clinicaltrials.gov/ct2/show/NCT02906800).

The traditional Chinese medicine extracted from the *Bufo* toad, HuaChanSu, is known to exert antitumor effects. In a pilot study involving patients with NSCLC, hepatocellular carcinoma, and pancreatic cancer, this extract was well tolerated and promoted disease stabilization. In a phase II clinical trial, the safety profile and the therapeutic potential of HuaChanSu combined with gemcitabine were investigated in individuals affected by advanced or metastatic pancreatic cancer. In this trial, the extract was well tolerated, but failed in other aspects [3].

## 5. Future Perspectives and Conclusions

In recent years, many studies have attempted to determine and understand the anticancer potential of CGs. Advances in treatment strategies, including combination with chemotherapy or immunotherapy, may improve the observed anticancer effects and consequently the average survival of cancer patients. Therefore, further investigation will allow the positioning of CGs as multimodal drug candidates. Moreover, combination treatment strategies and targeted delivery of CGs may further improve outcomes.

A promising translational research area is the development of combination strategies that use CGs together with chemotherapeutic agents. This approach may involve the administration of low subtoxic doses of CGs to prevent cardiotoxicity combined with selected broad or targeted anticancer agents. Moreover, this approach could lead to the stratification of specific subgroups of patients shown to benefit from such combinations. Recently, a synergistic effect was observed after combining the Mcl-1-targeting the cardenolide UNBS1450, with the BH3-mimetic agent, ABT-199, in Mcl-1-overexpressing forms of AML. This combination treatment was safe for the platelet, lymphocyte, and healthy CD34^+^ stem cells. This study, involving 23 patient samples, allowed the authors to identify subgroups of patients who were unresponsive to ABT-199 treatment alone, but responded well to a combination of both compounds [88].

In addition to the new mechanisms, the delivery method of CGs may be an important issue to explore. Novel theranostic approaches could allow the use of CGs in cancer treatment by improving efficacy and reducing toxicity, mainly for CGs that present a narrow therapeutic index. As an example, an octreotide–periplocymarin (**15**) conjugate prodrug was described to deliver this cardenolide to the tumor cells via somatostatin receptor subtypes. In radiation oncology, this conjugate was more cytotoxic to MCF-7 and HepG2 tumor cells, but less cytotoxic to L-02 non-tumor cells. Tissue distribution of the conjugate using the H22 tumor model in mice resulted in a greater accumulation in tumors and a lower distribution in heart and liver compared to periplocymarin (**15**) alone [89]. Another study that sought to improve the delivery of CGs to reduce cardiotoxicity was performed with acetylthevetin B (**16**), isolated from the seeds of *Thevetia peruviana* (Pers), which showed potent in vitro cytotoxic activity. To improve its action, acetylthevetin B was encapsulated in chitosan-pluronic P123 and was more cytotoxic to A549 lung cancer cells than the cardenolide alone. The accumulation of acetylthevetin B-loaded micelles in vivo, in the tumor and in lungs from mice was higher than that of free acetylthevetin B. There were no pathological changes to other tissues [90]. These delivery strategies exhibited tumor selectivity and may be a future targeting approach used to improve the safety profile of CGs for cancer therapy.

Another promising approach is the delivery of drugs using nanoparticles (NPs) in order to decrease side effects. NPs can deliver drugs into tumor cells with minimal drug without affecting normal cells. Conjugation of NPs with ligands of cancer-specific tumor biomarkers is a potent therapeutic approach [91,92]. A targeted moiety conjugated to the surface of NPs can ensure their delivery to the target site by receptor-mediated recognition. Integrin α_v_β_3_ is specifically overexpressed by endothelial vascular cells contributing to tumor growth; conversely, α_v_β_3_ integrin is not expressed by normal tissues. In this way, α_v_β_3_ integrin is an ideal target molecule. Since arginine-glycine-aspartic acid (RGD) peptides can effectively bind to α_v_β_3_ integrins, it serves as basis for active cancer targeting via NPs [93]. For this reason, NPs of methoxy polyethylene glycol (mPEG), polylactic-*co*-glycolic acid (PLGA), poly-L-lysine (PLL), and cyclic arginine-glycine-aspartic acid (cRGD) were loaded with bufalin (**17**) and tested in SW620 colon cancer-bearing mice. The cytotoxicity of the bufalin-loaded mPEG-PLGA-PLL-cRGD NPs was greater than that of bufalin alone. The NPs could be efficiently internalized in SW620 colon cancer cells. The targeting property of the cRGD moiety was proven by cell uptake and an in vivo target study. These results implied that the mPEG-PLGA-PLL-cRGD NPs enhanced the in vivo therapeutic efficacy [93]. Another way to deliver bufalin was using bovine serum albumin (BSA) nanoparticles. The tumor inhibition effect of bufalin-loaded BSA nanoparticles was stronger than that of bufalin alone in vivo. In this way, bufalin-loaded BSA nanoparticles seem to be a promising liver-targeted drug delivery system with a high liver uptake and a strong antitumor activity against hepatocellular carcinoma [94].

Several new approaches should be considered to deliver CGs in tumors by sparing healthy cells. For that, antibodies or ligands can be used to target antigens that are specific to cancer cells, called tumor-specific antigens, or to target antigens overexpressed by cancer cells, called tumor-associated antigens. Even if all targeting therapies should be considered to reduce cytotoxicity, the targeting with non-antibody ligands, such as arginine–glycine–aspartate (RGD), folate, transferrin, etc., present an important disadvantage since these ligands are abundantly present, even on normal cells, and hence the cytotoxic agent targeted through these ligands can bind to non-target tissues. Several technologies using this approach were developed, such as antibody-directed enzyme prodrug therapy, antibody cytokine fusion proteins, antibody and cancer vaccine targeting, antibody ligand-fused proteins, and antibodies for targeted siRNA delivery [95]. These possibilities can open the frontiers to overcome the undesirable effects of CGS.

Altogether, the data obtained until now strongly suggest that CGs are potent chemo- and immunotherapeutic agents. Multiple excellent and innovative strategies have been evaluated and several studies have shown that CGs are selective and safe, and favor future CG drug development.

## Figures and Tables

**Table 1:** In vitro CGs molecular target pathways in cancer cells.

Compound	Cell Line	Molecular Target and Mechanism of Action	Reference
OuaCGsbain (**1**)	**HeLa**	- ROCK activation - membrane blebbing - cell death	[57]
	**A549 and H1975**	- Autophagic cell death via JNK-dependent decrease of Bcl-2 expression	[58]
	**DAOY**	- Inhibition of EGF signaling	[59]
Digoxin (**2**)	**HeLa**	- Down-regulation of SRSF3	[60]
Digitoxin (**3**)	**GSC**	- Attenuation of hypoxia-induced VEGF expression - Suppression of HIF-1α accumulation	[61]
Lanatoside C (**5**)	**Huh7 and Mahlavu**	- Induction of cell death independent of the PTEN status	[62]
Oleandrin (**7**)	**U2OS and SaOS-2**	- Suppression of Wnt/β-catenin	[63]
Convallatoxin (**11**)	**HCT116**	- Antiproliferative effects leading to cell death independent of p53	[64]
Bufalin (**17**)	**MCF-7**	- Inhibition of SRC-3 and SRC-1	[65]
	**H1975**	- Induced apoptosis via downregulation of Mcl-1 by GSK-3β activation	[66]
	**BxPC3-luc2**	- Induced cell cycle arrest via the c-Myc/NF-κB pathway	[67]

Cell lines: Hela, cervical cancer; A549 and H1975, non-small-cell lung cancer; DAOY: human medulloblastoma; GSC, human glioma stem cells; Huh7 and Mahlavu, human hepatocellular carcinoma; U2OS and SaOS-2; osteosarcoma; HCT116, human colon cancer; MCF-7: breast cancer; BxPC-3-luc2: pancreatic adenocarcinoma; Abbreviations: Bcl-2, B-cell lymphoma-2; EGF, epidermal growth factor; GSK-3β, glycogen synthase kinase-3β; HIF-1α, hypoxia inducible factor-1α; JNK, c-Jun N-terminal kinase; Mcl-1, myeloid cell leukemia-1; NF-κB, nuclear factor kappa B; PTEN, Phosphatase and tensin homolog; ROCK, Rho-associated protein kinase; SRC, sarcoma; SRSF3, serine/arginine-rich splicing factor 3, Serine/arginine-rich splicing factor-3; vascular endothelial growth factor, VEGF; Wnt, wingless.
